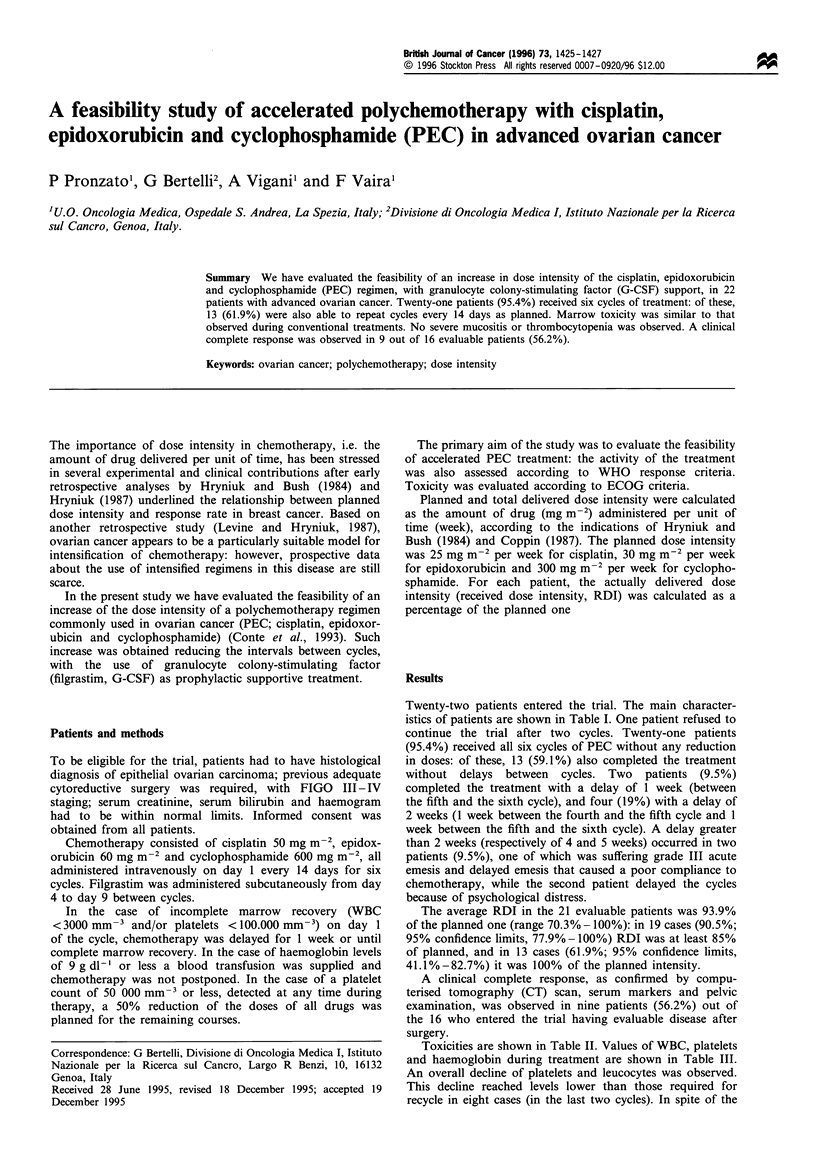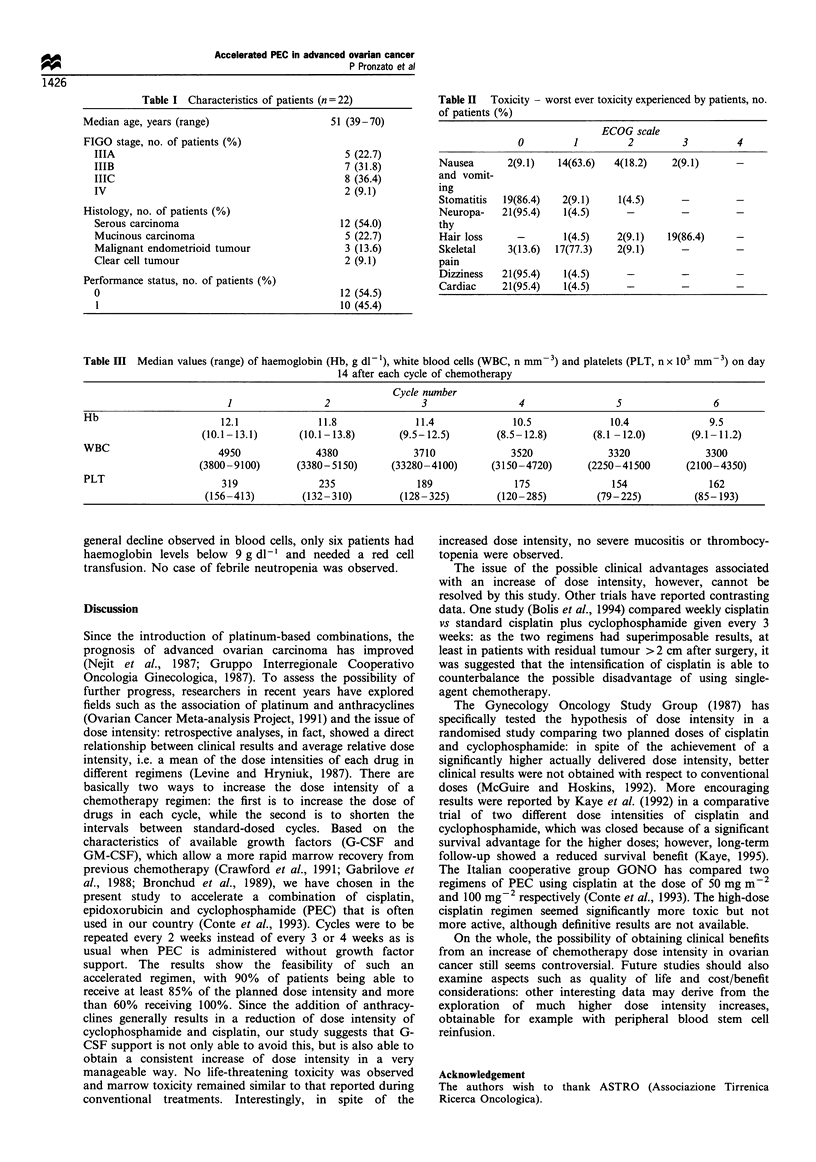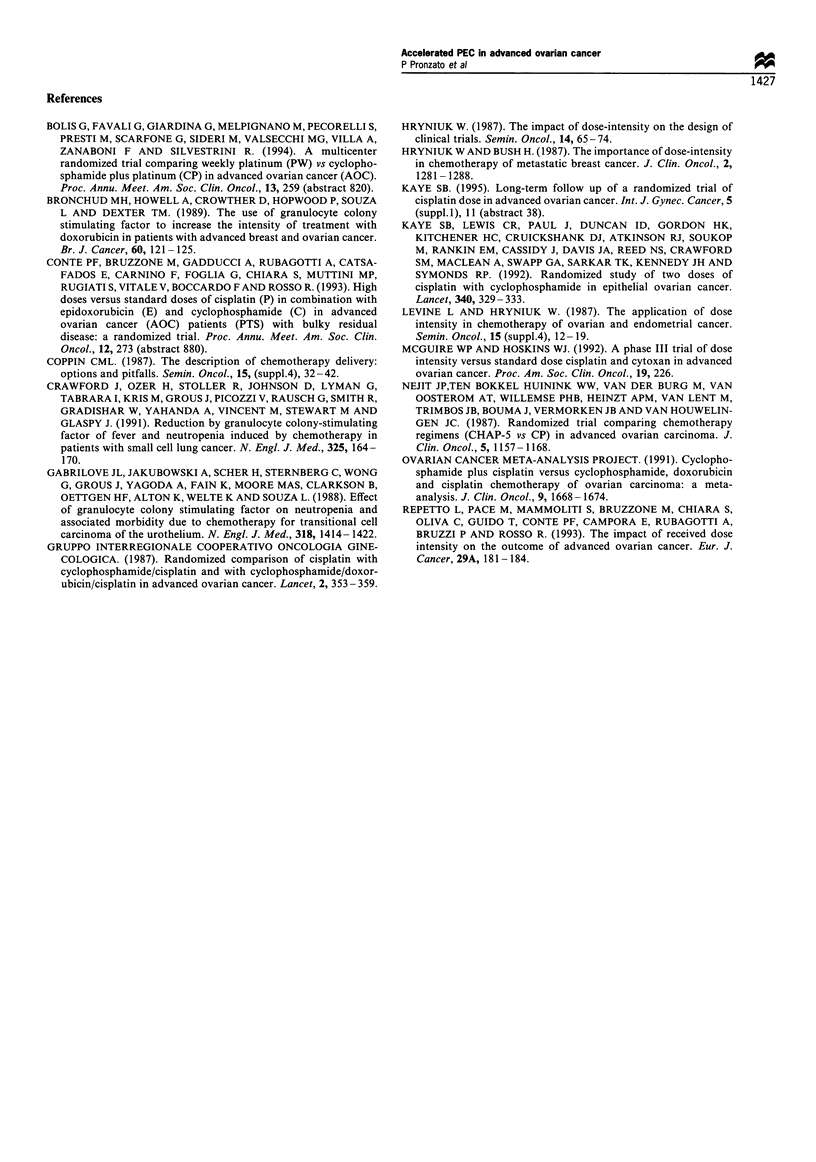# A feasibility study of accelerated polychemotherapy with cisplatin, epidoxorubicin and cyclophosphamide (PEC) in advanced ovarian cancer.

**DOI:** 10.1038/bjc.1996.270

**Published:** 1996-06

**Authors:** P. Pronzato, G. Bertelli, A. Vigani, F. Vaira

**Affiliations:** U.O. Oncologia Medica, Ospedale S. Andrea, La Spezia, Italy.

## Abstract

We have evaluated the feasibility of an increase in dose intensity of the cisplatin, epidoxorubicin and cyclophosphamide (PEC) regimen, with granulocyte colony-stimulating factor (G-CSF) support, in 22 patients with advanced ovarian cancer. Twenty-one patients (95.4%) received six cycles of treatment: of these, 13 (61.9%) were also able to repeat cycles every 14 days as planned. Marrow toxicity was similar to that observed during conventional treatments. No severe mucositis or thrombocytopenia was observed. A clinical complete response was observed in 9 out of 16 evaluable patients (56.2%).


British Journal of Cancer (1996) 73, 1425-1427
© 1996 Stockton Press All rights reserved 0007-0920/96 $12.00

### A feasibility study of accelerated polychemotherapy with cisplatin, epidoxorubicin and cyclophosphamide (PEC) in advanced ovarian cancer

P Pronzato[1], G Bertelli[2], A Vigani[1] and F Vaira[1]

[1]U.O. Oncologia Medica, Ospedale S. Andrea, La Spezia, Italy; [2]Divisione di Oncologia Medica I, Istituto Nazionale per la Ricerca sul Cancro, Genoa, Italy.

**Summary** We have evaluated the feasibility of an increase in dose intensity of the cisplatin, epidoxorubicin and cyclophosphamide (PEC) regimen, with granulocyte colony-stimulating factor (G-CSF) support, in 22 patients with advanced ovarian cancer. Twenty-one patients (95.4%) received six cycles of treatment: of these, 13 (61.9%) were also able to repeat cycles every 14 days as planned. Marrow toxicity was similar to that observed during conventional treatments. No severe mucositis or thrombocytopenia was observed. A clinical complete response was observed in 9 out of 16 evaluable patients (56.2%).

**Keywords:** ovarian cancer; polychemotherapy; dose intensity

The importance of dose intensity in chemotherapy, i.e. the amount of drug delivered per unit of time, has been stressed in several experimental and clinical contributions after early retrospective analyses by Hryniuk and Bush (1984) and Hryniuk (1987) underlined the relationship between planned dose intensity and response rate in breast cancer. Based on another retrospective study (Levine and Hryniuk, 1987), ovarian cancer appears to be a particularly suitable model for intensification of chemotherapy: however, prospective data about the use of intensified regimens in this disease are still scarce.

In the present study we have evaluated the feasibility of an increase of the dose intensity of a polychemotherapy regimen commonly used in ovarian cancer (PEC; cisplatin, epidoxorubicin and cyclophosphamide) (Conte *et al.*, 1993). Such increase was obtained reducing the intervals between cycles, with the use of granulocyte colony-stimulating factor (filgrastim, G-CSF) as prophylactic supportive treatment.

### Patients and methods

To be eligible for the trial, patients had to have histological diagnosis of epithelial ovarian carcinoma; previous adequate cytoreductive surgery was required, with FIGO III-IV staging; serum creatinine, serum bilirubin and haemogram had to be within normal limits. Informed consent was obtained from all patients.

Chemotherapy consisted of cisplatin 50 mg $m^{-2}$, epidoxorubicin 60 mg $m^{-2}$ and cyclophosphamide 600 mg $m^{-2}$, all administered intravenously on day 1 every 14 days for six cycles. Filgrastim was administered subcutaneously from day 4 to day 9 between cycles.

In the case of incomplete marrow recovery (WBC $<3000$ $mm^{-3}$ and/or platelets $<100.000$ $mm^{-3}$) on day 1 of the cycle, chemotherapy was delayed for 1 week or until complete marrow recovery. In the case of haemoglobin levels of 9 g $dl^{-1}$ or less a blood transfusion was supplied and chemotherapy was not postponed. In the case of a platelet count of 50 000 $mm^{-3}$ or less, detected at any time during therapy, a 50% reduction of the doses of all drugs was planned for the remaining courses.

The primary aim of the study was to evaluate the feasibility of accelerated PEC treatment: the activity of the treatment was also assessed according to WHO response criteria. Toxicity was evaluated according to ECOG criteria.

Planned and total delivered dose intensity were calculated as the amount of drug (mg $m^{-2}$) administered per unit of time (week), according to the indications of Hryniuk and Bush (1984) and Coppin (1987). The planned dose intensity was 25 mg $m^{-2}$ per week for cisplatin, 30 mg $m^{-2}$ per week for epidoxorubicin and 300 mg $m^{-2}$ per week for cyclophosphamide. For each patient, the actually delivered dose intensity (received dose intensity, RDI) was calculated as a percentage of the planned one

### Results

Twenty-two patients entered the trial. The main characteristics of patients are shown in Table I. One patient refused to continue the trial after two cycles. Twenty-one patients (95.4%) received all six cycles of PEC without any reduction in doses: of these, 13 (59.1%) also completed the treatment without delays between cycles. Two patients (9.5%) completed the treatment with a delay of 1 week (between the fifth and the sixth cycle), and four (19%) with a delay of 2 weeks (1 week between the fourth and the fifth cycle and 1 week between the fifth and the sixth cycle). A delay greater than 2 weeks (respectively of 4 and 5 weeks) occurred in two patients (9.5%), one of which was suffering grade III acute emesis and delayed emesis that caused a poor compliance to chemotherapy, while the second patient delayed the cycles because of psychological distress.

The average RDI in the 21 evaluable patients was 93.9% of the planned one (range 70.3% - 100%): in 19 cases (90.5%; 95% confidence limits, 77.9% - 100%) RDI was at least 85% of planned, and in 13 cases (61.9%; 95% confidence limits, 41.1% - 82.7%) it was 100% of the planned intensity.

A clinical complete response, as confirmed by computerised tomography (CT) scan, serum markers and pelvic examination, was observed in nine patients (56.2%) out of the 16 who entered the trial having evaluable disease after surgery.

Toxicities are shown in Table II. Values of WBC, platelets and haemoglobin during treatment are shown in Table III. An overall decline of platelets and leucocytes was observed. This decline reached levels lower than those required for recycle in eight cases (in the last two cycles). In spite of the

Correspondence: G Bertelli, Divisione di Oncologia Medica I, Istituto Nazionale per la Ricerca sul Cancro, Largo R Benzi, 10, 16132 Genoa, Italy
Received 28 June 1995, revised 18 December 1995; accepted 19 December 1995

**Table I** Characteristics of patients ($n = 22$)

| | |
|---|---|
| Median age, years (range) | 51 (39–70) |
| FIGO stage, no. of patients (%) | |
| IIIA | 5 (22.7) |
| IIIB | 7 (31.8) |
| IIIC | 8 (36.4) |
| IV | 2 (9.1) |
| Histology, no. of patients (%) | |
| Serous carcinoma | 12 (54.0) |
| Mucinous carcinoma | 5 (22.7) |
| Malignant endometrioid tumour | 3 (13.6) |
| Clear cell tumour | 2 (9.1) |
| Performance status, no. of patients (%) | |
| 0 | 12 (54.5) |
| 1 | 10 (45.4) |

**Table II** Toxicity – worst ever toxicity experienced by patients, no. of patients (%)

| | *ECOG scale* | | | | |
|---|---|---|---|---|---|
| | *0* | *1* | *2* | *3* | *4* |
| Nausea and vomiting | 2(9.1) | 14(63.6) | 4(18.2) | 2(9.1) | – |
| Stomatitis | 19(86.4) | 2(9.1) | 1(4.5) | – | – |
| Neuropathy | 21(95.4) | 1(4.5) | – | – | – |
| Hair loss | – | 1(4.5) | 2(9.1) | 19(86.4) | – |
| Skeletal pain | 3(13.6) | 17(77.3) | 2(9.1) | – | – |
| Dizziness | 21(95.4) | 1(4.5) | – | – | – |
| Cardiac | 21(95.4) | 1(4.5) | – | – | – |

**Table III** Median values (range) of haemoglobin (Hb, g $dl^{-1}$), white blood cells (WBC, n $mm^{-3}$) and platelets (PLT, n $\times 10^3$ $mm^{-3}$) on day 14 after each cycle of chemotherapy

| | *Cycle number* | | | | | |
|---|---|---|---|---|---|---|
| | *1* | *2* | *3* | *4* | *5* | *6* |
| Hb | 12.1 (10.1–13.1) | 11.8 (10.1–13.8) | 11.4 (9.5–12.5) | 10.5 (8.5–12.8) | 10.4 (8.1 –12.0) | 9.5 (9.1–11.2) |
| WBC | 4950 (3800–9100) | 4380 (3380–5150) | 3710 (33280–4100) | 3520 (3150–4720) | 3320 (2250–41500 | 3300 (2100–4350) |
| PLT | 319 (156–413) | 235 (132–310) | 189 (128–325) | 175 (120–285) | 154 (79–225) | 162 (85–193) |

general decline observed in blood cells, only six patients had haemoglobin levels below 9 g $dl^{-1}$ and needed a red cell transfusion. No case of febrile neutropenia was observed.

### Discussion

Since the introduction of platinum-based combinations, the prognosis of advanced ovarian carcinoma has improved (Nejit *et al.*, 1987; Gruppo Interregionale Cooperativo Oncologia Ginecologica, 1987). To assess the possibility of further progress, researchers in recent years have explored fields such as the association of platinum and anthracyclines (Ovarian Cancer Meta-analysis Project, 1991) and the issue of dose intensity: retrospective analyses, in fact, showed a direct relationship between clinical results and average relative dose intensity, i.e. a mean of the dose intensities of each drug in different regimens (Levine and Hryniuk, 1987). There are basically two ways to increase the dose intensity of a chemotherapy regimen: the first is to increase the dose of drugs in each cycle, while the second is to shorten the intervals between standard-dosed cycles. Based on the characteristics of available growth factors (G-CSF and GM-CSF), which allow a more rapid marrow recovery from previous chemotherapy (Crawford *et al.*, 1991; Gabrilove *et al.*, 1988; Bronchud *et al.*, 1989), we have chosen in the present study to accelerate a combination of cisplatin, epidoxorubicin and cyclophosphamide (PEC) that is often used in our country (Conte *et al.*, 1993). Cycles were to be repeated every 2 weeks instead of every 3 or 4 weeks as is usual when PEC is administered without growth factor support. The results show the feasibility of such an accelerated regimen, with 90% of patients being able to receive at least 85% of the planned dose intensity and more than 60% receiving 100%. Since the addition of anthracyclines generally results in a reduction of dose intensity of cyclophosphamide and cisplatin, our study suggests that G-CSF support is not only able to avoid this, but is also able to obtain a consistent increase of dose intensity in a very manageable way. No life-threatening toxicity was observed and marrow toxicity remained similar to that reported during conventional treatments. Interestingly, in spite of the increased dose intensity, no severe mucositis or thrombocytopenia were observed.

The issue of the possible clinical advantages associated with an increase of dose intensity, however, cannot be resolved by this study. Other trials have reported contrasting data. One study (Bolis *et al.*, 1994) compared weekly cisplatin *vs* standard cisplatin plus cyclophosphamide given every 3 weeks: as the two regimens had superimposable results, at least in patients with residual tumour >2 cm after surgery, it was suggested that the intensification of cisplatin is able to counterbalance the possible disadvantage of using single-agent chemotherapy.

The Gynecology Oncology Study Group (1987) has specifically tested the hypothesis of dose intensity in a randomised study comparing two planned doses of cisplatin and cyclophosphamide: in spite of the achievement of a significantly higher actually delivered dose intensity, better clinical results were not obtained with respect to conventional doses (McGuire and Hoskins, 1992). More encouraging results were reported by Kaye *et al.* (1992) in a comparative trial of two different dose intensities of cisplatin and cyclophosphamide, which was closed because of a significant survival advantage for the higher doses; however, long-term follow-up showed a reduced survival benefit (Kaye, 1995). The Italian cooperative group GONO has compared two regimens of PEC using cisplatin at the dose of 50 mg $m^{-2}$ and 100 $mg^{-2}$ respectively (Conte *et al.*, 1993). The high-dose cisplatin regimen seemed significantly more toxic but not more active, although definitive results are not available.

On the whole, the possibility of obtaining clinical benefits from an increase of chemotherapy dose intensity in ovarian cancer still seems controversial. Future studies should also examine aspects such as quality of life and cost/benefit considerations: other interesting data may derive from the exploration of much higher dose intensity increases, obtainable for example with peripheral blood stem cell reinfusion.

**Acknowledgement**
The authors wish to thank ASTRO (Associazione Tirrenica Ricerca Oncologica).

### References

BOLIS G, FAVALI G, GIARDINA G, MELPIGNANO M, PECORELLI S, PRESTI M, SCARFONE G, SIDERI M, VALSECCHI MG, VILLA A, ZANABONI F AND SILVESTRINI R. (1994). A multicenter randomized trial comparing weekly platinum (PW) *vs* cyclophosphamide plus platinum (CP) in advanced ovarian cancer (AOC). *Proc. Annu. Meet. Am. Soc. Clin. Oncol.*, **13,** 259 (abstract 820).

BRONCHUD MH, HOWELL A, CROWTHER D, HOPWOOD P, SOUZA L AND DEXTER TM. (1989). The use of granulocyte colony stimulating factor to increase the intensity of treatment with doxorubicin in patients with advanced breast and ovarian cancer. *Br. J. Cancer*, **60,** 121–125.

CONTE PF, BRUZZONE M, GADDUCCI A, RUBAGOTTI A, CATSAFADOS E, CARNINO F, FOGLIA G, CHIARA S, MUTTINI MP, RUGIATI S, VITALE V, BOCCARDO F AND ROSSO R. (1993). High doses versus standard doses of cisplatin (P) in combination with epidoxorubicin (E) and cyclophosphamide (C) in advanced ovarian cancer (AOC) patients (PTS) with bulky residual disease: a randomized trial. *Proc. Annu. Meet. Am. Soc. Clin. Oncol.*, **12,** 273 (abstract 880).

COPPIN CML. (1987). The description of chemotherapy delivery: options and pitfalls. *Semin. Oncol.*, **15,** (suppl.4), 32–42.

CRAWFORD J, OZER H, STOLLER R, JOHNSON D, LYMAN G, TABRARA I, KRIS M, GROUS J, PICOZZI V, RAUSCH G, SMITH R, GRADISHAR W, YAHANDA A, VINCENT M, STEWART M AND GLASPY J. (1991). Reduction by granulocyte colony-stimulating factor of fever and neutropenia induced by chemotherapy in patients with small cell lung cancer. *N. Engl. J. Med.*, **325,** 164–170.

GABRILOVE JL, JAKUBOWSKI A, SCHER H, STERNBERG C, WONG G, GROUS J, YAGODA A, FAIN K, MOORE MAS, CLARKSON B, OETTGEN HF, ALTON K, WELTE K AND SOUZA L. (1988). Effect of granulocyte colony stimulating factor on neutropenia and associated morbidity due to chemotherapy for transitional cell carcinoma of the urothelium. *N. Engl. J. Med.*, **318,** 1414–1422.

GRUPPO INTERREGIONALE COOPERATIVO ONCOLOGIA GINECOLOGICA. (1987). Randomized comparison of cisplatin with cyclophosphamide/cisplatin and with cyclophosphamide/doxorubicin/cisplatin in advanced ovarian cancer. *Lancet*, **2,** 353–359.

HRYNIUK W. (1987). The impact of dose-intensity on the design of clinical trials. *Semin. Oncol.*, **14,** 65–74.

HRYNIUK W AND BUSH H. (1987). The importance of dose-intensity in chemotherapy of metastatic breast cancer. *J. Clin. Oncol.*, **2,** 1281–1288.

KAYE SB. (1995). Long-term follow up of a randomized trial of cisplatin dose in advanced ovarian cancer. *Int. J. Gynec. Cancer*, **5** (suppl.1), 11 (abstract 38).

KAYE SB, LEWIS CR, PAUL J, DUNCAN ID, GORDON HK, KITCHENER HC, CRUICKSHANK DJ, ATKINSON RJ, SOUKOP M, RANKIN EM, CASSIDY J, DAVIS JA, REED NS, CRAWFORD SM, MACLEAN A, SWAPP GA, SARKAR TK, KENNEDY JH AND SYMONDS RP. (1992). Randomized study of two doses of cisplatin with cyclophosphamide in epithelial ovarian cancer. *Lancet*, **340,** 329–333.

LEVINE L AND HRYNIUK W. (1987). The application of dose intensity in chemotherapy of ovarian and endometrial cancer. *Semin. Oncol.*, **15** (suppl.4), 12–19.

MCGUIRE WP AND HOSKINS WJ. (1992). A phase III trial of dose intensity versus standard dose cisplatin and cytoxan in advanced ovarian cancer. *Proc. Am. Soc. Clin. Oncol.*, **19,** 226.

NEJIT JP,TEN BOKKEL HUININK WW, VAN DER BURG M, VAN OOSTEROM AT, WILLEMSE PHB, HEINZT APM, VAN LENT M, TRIMBOS JB, BOUMA J, VERMORKEN JB AND VAN HOUWELINGEN JC. (1987). Randomized trial comparing chemotherapy regimens (CHAP-5 *vs* CP) in advanced ovarian carcinoma. *J. Clin. Oncol.*, **5,** 1157–1168.

OVARIAN CANCER META-ANALYSIS PROJECT. (1991). Cyclophosphamide plus cisplatin versus cyclophosphamide, doxorubicin and cisplatin chemotherapy of ovarian carcinoma: a meta-analysis. *J. Clin. Oncol.*, **9,** 1668–1674.

REPETTO L, PACE M, MAMMOLITI S, BRUZZONE M, CHIARA S, OLIVA C, GUIDO T, CONTE PF, CAMPORA E, RUBAGOTTI A, BRUZZI P AND ROSSO R. (1993). The impact of received dose intensity on the outcome of advanced ovarian cancer. *Eur. J. Cancer*, **29A,** 181–184.